# To Clot or Not: HELLP Syndrome and Disseminated Intravascular Coagulation in an Eclamptic Patient with Intrauterine Fetal Demise

**DOI:** 10.1155/2020/9642438

**Published:** 2020-07-07

**Authors:** Justin Walker, Anthony Bonavia

**Affiliations:** ^1^Department of Anesthesiology and Perioperative Medicine, Penn State Health Milton S. Hershey Medical Center, 500 University Dr., Mailcode H187, Hershey, PA 17033, USA; ^2^Department of Pharmacology, Penn State College of Medicine, 700 HMC Crescent Road, Hershey, PA 17033, USA

## Abstract

A 39-year-old G2P1001 female presented from an outside hospital following an eclamptic seizure in the setting of HELLP syndrome. This condition was complicated by intrauterine fetal demise and disseminated intravascular coagulation, which required an emergent cesarean section. We report the work-up and intraoperative and postoperative management of this complex patient with multiple medical needs. We focus on the hemostatic abnormalities in this patient and describe how our management would differ from that of a similar, nonpregnant patient.

## 1. Introduction

Disseminated intravascular coagulation (DIC) is a condition characterized by the uncontrolled activation of the hemostatic system, resulting in widespread microvascular thrombosis, ischemic end-organ dysfunction, and the rapid consumption of coagulation factors with uncontrolled bleeding [1–3]. In obstetric patients, DIC may result from (1) acute peripartum hemorrhage, (2) placental abruption, (3) preeclampsia/eclampsia/HELLP (hemolysis, elevated liver enzymes, low platelets) syndrome, (4) retained stillbirth, (5) septic abortion, (6) amniotic fluid embolism, and/or (7) acute fatty liver of pregnancy [1, 4]. Although the prompt diagnosis and management of DIC is paramount to optimizing maternal outcomes, normal changes in the coagulation profile of pregnant patients may delay diagnosis and increase maternal mortality [2, 5–8]. Immediate management of DIC and HELLP syndrome is primarily focused on blood product transfusion and coagulation factor repletion to facilitate hemostasis. However, resolution of these conditions requires treatment of the precipitating cause [1, 3, 9, 10].

We report the case of a 39-year-old patient with eclampsia, HELLP syndrome, and DIC, suspected to be secondary to retained products of conception, who required emergent fetal delivery. We discuss this patient's diagnosis, intraoperative management, fluid resuscitation, and intensive care course and, then, review the pertinent medical literature surrounding these conditions.

## 2. Case Presentation

A 39-year-old G2P1001 female, with no significant past medical history, presented at 33 0/7 weeks of gestational age following two eclamptic seizures, the first occurring at home and second, en route to the hospital. Her antenatal course had been unremarkable. Upon hospital presentation, she was found to have intrauterine fetal demise (IUFD) and trace subarachnoid hemorrhage and radiographic evidence of posterior reversible encephalopathy syndrome (PRES). The patient's clinical presentation was remarkable for thrombocytopenia (38,000 platelets/microliter), a systolic blood pressure of greater than 140 mmHg, and amnesia to the day's events likely secondary to her postictal state. She was additionally noted to have moderate vaginal bleeding at the time of presentation. She was immediately transferred to our tertiary care center intensive care unit (ICU) for further medical management in the setting of suspected HELLP syndrome.

Upon arrival, she received intravenous magnesium sulfate, and a radial arterial line was established due to the need for frequent blood analysis. Initial results confirmed thrombocytopenia (21,000 platelets/microliter), transaminitis (ALT 2,639 units/L, AST 5,875 units/L), and hyperbilirubinemia (bilirubin 6.6 mg/dL). The patient was also coagulopathic with an INR 2.4, PT 26 s (9.7 (8.6–12.4)), PTT 39 s (29.0 s (25.6–34.9)), and fibrinogen 0.83 g/L (4.18 g/L (2.79–5.91)) and had an increased concentration of fibrin split products (>20 mcg/mL). Her pregnancy-modified disseminated intravascular coagulation (DIC) score was 51, which was consistent with a high risk of DIC.

Within 3 hours of hospital admission, the patient's serum hemoglobin concentration had decreased from 12.5 mg/dL to 9.1 mg/dL, and the patient was emergently taken to the operating room for delivery of the nonviable fetus. She underwent an uneventful rapid sequence endotracheal intubation followed by the placement of a right internal jugular multilumen access catheter to facilitate intraoperative resuscitation. She underwent cesarean section which was complicated by uterine atony. Oxytocin (20 units administered intravenously, twice) and carboprost (250 mcg intramuscular injection, administered three times) were administered. Massive hemorrhage ensued, requiring bilateral uterine artery ligation followed by total abdominal hysterectomy. She received 6 units of packed red blood cells, 6 units fresh-frozen plasma, and 5 units of platelets, as well as cryoprecipitate, fibrinogen concentrate, and a 4-factor prothrombin complex concentrate. Additionally, 1000 mg tranexamic acid was given as an infusion over 10 minutes at the beginning of the case. The total estimated blood loss during the cesarean section was 3 liters. In spite of significant blood loss, her mean arterial pressures throughout the case ranged from 81 mmHg to 127 mmHg. At the termination of surgery (duration 290 minutes), she was felt to be adequately resuscitated, hemodynamically stable, and adequately recovered from the neuromuscular blockade, and she was, thus, extubated successfully in the operating room. She was admitted to the ICU postoperatively for further monitoring and management.

Lab values on arrival to the ICU demonstrated persistent acute blood loss anemia, requiring an additional 4 units of packed red blood cells within the first 12 hours following surgery. Thromboelastography (TEG, Kaolin assay) 4 hours postoperatively demonstrated a clotting time of 9.9 minutes (4–8 minutes) and an alpha angle of 51.3 degrees (47–74 degrees). This corresponded to a serum fibrinogen level of 2.66 g/L. A follow-up TEG was performed 20 hours postoperatively and demonstrated a clotting time of 10.5 minutes with an angle of 45.7 degrees. Corresponding PT, aPTT, INR, and fibrinogen levels all improved. Her fibrin split products remained elevated (>20 mcg/mL) for 24 hours postoperatively, but were not continually trended. Since the patient's hemoglobin and clinical status had stabilized, no additional blood transfusions were deemed necessary. Her aPTT and PT normalized at 27 hours and 38 hours postoperatively, respectively. Thromboprophylaxis (subcutaneous heparin 5000 U, every 8 hours) was initiated on postoperative day 3.

**Figure 1:**
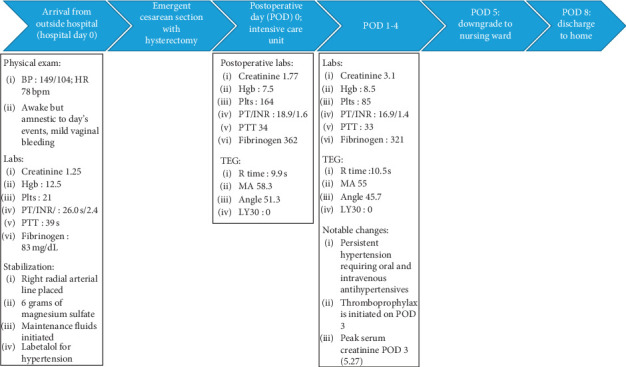
Time course flow-chart of the most significant events.

The patient's postoperative course was remarkable for hypertension, requiring a combination of oral and intravenous antihypertensive agents and oliguric acute kidney injury secondary to ischemic acute tubular necrosis with a peak creatinine value of 5.27 mg/dL. A summary of these events are available in Figure 1.

The patient's kidney function continued to improve with medical management, and the patient was transitioned from the ICU to the nursing ward on hospital day 5 and discharged home on day 8. Upon follow-up, she reported fatigue, headaches, and neurocognitive difficulties with concentration and task switching. At the time of writing, 14 weeks after her hospitalization, these complications have kept her from returning to work, and she is currently undergoing neuropsychological testing which, to date, has demonstrated difficulty with vigilance on automated attention measures, as well as a mild decline in word list recall. A repeat MRI did not demonstrate any residual abnormalities, consistent with resolving PRES.

## 3. Discussion

DIC is a clinical diagnosis and a rare complication of pregnancy, with a reported incidence of 0.03% to 0.35% [6, 11]. In the gravid patient, it is most commonly seen as a complication of placental abruption or postpartum hemorrhage. It may also be seen in HELLP syndrome or with retained products of conception [4, 8]. DIC has classically been broken into clinical subtypes which include DIC with suppressed fibrinolysis (typical of sepsis), DIC with enhanced fibrinolysis (aortic aneurysms, hemorrhage, and abruption placentae), and DIC with balanced fibrinolysis (cancer) [12]. These subtypes may be differentiated based on symptoms and laboratory derangements. DIC with enhanced fibrinolysis demonstrates symptoms of severe bleeding with elevated D-dimer and only a slight increase in the plasminogen activator inhibitor. DIC with suppressed fibrinolysis tends to present with severe organ dysfunction in association with a mild elevation in D-dimer and markedly increased levels of plasminogen activator inhibitor. DIC associated with hemorrhage and intrauterine fetal demise is thought to be triggered by the exposure of tissue-factor on the vascular endothelium with results in the massive activation of the extrinsic coagulation cascade [13].

The diagnosis of DIC in pregnancy requires a high index of suspicion, due to various laboratory changes in the gravid patient which make its diagnosis more challenging (Table 1). These changes include hyperfibrinogenemia (normal 3^rd^ trimester value of approximately 5 g/L), decreased prothrombin (PT) and partial thromboplastin times (aPTT), and thrombocytopenia [1, 4, 5, 14, 15]. Therefore, hematologic laboratory results that align with normal values in the nongravid patient should elicit additional scrutiny by the clinician. While fibrin split products are often used as an additional marker of DIC, these values are commonly elevated in the gravid patient, and therefore, they should not be used exclusively for diagnosing DIC in this population. Clinical signs that are characteristic of DIC include bleeding from catheterization sites, mucocutaneous bleeding, or scattered petechiae [16]. Specific obstetric conditions such as acute fatty liver of pregnancy (AFLP) and HELLP syndrome further complicate the diagnosis of DIC, as hepatic synthetic function is compromised and results in decreased levels of both anticoagulation and procoagulation blood components.

The original International Society of Thrombosis and Haemostasis (ISTH) scoring system for DIC [17] did not account for normal hemostatic changes of pregnancy. However, Erez et al. published a pregnancy-modified DIC scoring system which highlights three components of the ISTH DIC scoring system [6]. These are platelet count, fibrinogen concentration, and PT difference (defined as the difference between the patient's value and the laboratory normal value) [6, 17]. It should be noted that this study excluded patients with severe preeclampsia, but it did not exclude patients with HELLP syndrome.

This pregnancy-modified DIC scoring system has since been validated by Clark et al. [18]. Individually, for a platelet count ≤186 × 10^3^/*µ*L, fibrinogen concentration ≤3.9 g/L, and PT difference ≥1.55, the laboratory norm yielded sensitivities of 86%, 87%, and 87% and specificities of 71%, 92%, and 90%, respectively. Cutoff points for each of the three components were determined using receiver operator curves, and a weighted scoring system was created that outperformed each individual score. A value of ≥26 assigns a sensitivity of 88% and specificity of 96% for the diagnosis of DIC during pregnancy. Although a useful tool to assist in the recognition of DIC in pregnancy, the diagnosis of DIC is ultimately a clinical one, and physicians must be aware of the symptoms and risk-factors predisposing to the development of DIC.

The constituent breakdown of the pregnancy-modified DIC scoring system is presented in Table 1. The patient described above scored 51 when pregnancy-modified DIC scoring criteria were used (1 point for platelet count <50,000/L, 25 points for prothrombin time difference >1.5 normal value, and 25 points for fibrinogen level <3.0), corresponding to a very high probability of DIC. Although HELLP syndrome may have contributed to the coagulopathy and thrombocytopenia observed in our patient, hypofibrinogenemia (0.83 g/L) is atypical of HELLP syndrome alone. Given the limitation of the study by Erez et al., further studies that assess the validity of the pregnancy-modified DIC score in patients with concomitant HELLP syndrome should be performed, as the incidence of DIC in HELLP syndrome is reportedly as high as 12–15% [19, 20].

The management of DIC in the obstetric patient involves, in order of decreasing importance, (1) diagnosis and treatment of the causative underlying condition, (2) blood product transfusion and coagulation factor repletion, (3) regular clinical and laboratory reevaluation, and (4) involvement of appropriate specialists [1].

While the transfusion of blood products and clotting factors is required in these patients to treat acute hemorrhage, these interventions must be balanced with the concern of precipitating acute thrombosis [1, 5, 9, 10, 21]. Microvascular thrombosis may be manifest clinically as pulmonary emboli, venous thromboembolism, stroke, or acute tubular necrosis (ATN) resulting in acute kidney injury. In one study of gravid patients with DIC, there was a 6% incidence of ATN requiring dialysis [11]. In cases of DIC with thrombosis, therapeutic heparin may be considered. However, this is contraindicated in the setting of active hemorrhage or in patients with a high risk of bleeding, as is the case with parturients [22, 23]. Our institution's Massive Transfusion Protocol (MTP) was initiated in anticipation of severe hemorrhage. In spite of a balanced blood product transfusion, the patient experienced progressive hemorrhage, prompting the administration of 4-factor prothrombin complex concentrates (PCC) and tranexamic acid (TXA).

The use of PCC is controversial in DIC, as there is concern for PCC-induced acute thrombosis [3]. It should be noted that early studies addressing the risk of this complication occurred at a time when three-factor PCC (Factors II, IX, and X) was the mainstay of therapy. The pathophysiology of DIC is dependent on the tissue factor/Factor VII pathway, and thus, there is an early massive depletion of Factor VII in DIC. Newer four-factor and activated four-factor PCC include the addition of Factor VII, and a review by Franchini et al. demonstrated the utility of Factor VII in controlling hemorrhage in the obstetric patient with DIC [24, 25]. Theoretically, the inclusion of Factor VII may make four-factor PCC more suitable for the treatment of refractory hemorrhage in obstetric DIC. However, randomized controlled trials investigating this hypothesis are lacking.

Tranexamic acid (TXA) has seen a recent return to popularity in the trauma literature for acute hemorrhage. A 2017 multinational study of antifibrinolytic therapy for postpartum hemorrhage (WOMAN trial) demonstrated a survival benefit with the early administration of TXA [26–28]. TXA works by irreversibly blocking lysine-binding sites on plasminogen, thus preventing its conversion to plasmin and inhibiting fibrinolysis [24]. TXA use in DIC is controversial, and it must be used with extreme caution in DIC, as there is a risk of inducing microembolisms. The use of TXA may be indicated in DIC with enhanced fibrinolysis and severe hemorrhage, and the embolic risk should be weighed when considering its administration [12, 29–32]. In this case, the patient had evidence of hyperfibrinolysis with elevated fibrin degradation products (FDP), as well as profound uterine atony, thus prompting the administration of TXA. A conversation between anesthesiologist and surgeon is imperative to determine the need for TXA on a case-by-case basis, and should be reserved for cases of refractory hemorrhage with evidence of severe hyperfibrinolysis.

While serial measurements of PT and aPTT values have historically been used to guide resuscitation efforts in DIC, recent literature suggests that a TEG may allow for a more targeted approach to prohemostatic interventions and allow for improved subclassification of DIC [5]. TEG provides a rapid and quantitative evaluation of all phases of hemostasis (clot initiation, propagation, strength, and dissolution), thus providing the anesthesiologist with data regarding the most likely source of coagulation defect(s) and the need for specific blood components for correction [33]. Normal reference values are available for pregnant patients (Table 2), which can help guide the application of TEG in these patients and mitigate some of the aforementioned shortcomings of PT and aPTT values [5, 34–38]. Specific information is available on normal values and repletion strategies but is outside the scope of this article [5, 39, 40]. The use of TEG to guide resuscitation efforts has been shown in both the trauma and obstetric literature to reduce the number of blood product transfusions in select populations, thereby reducing the risks of transfusion-associated morbidity [33, 41]. In the setting of a gravid patient with DIC and HELLP and/or acute fatty liver of pregnancy, TEG may be more appropriate for the trending of a patient's coagulation status and should be considered an adjunct to routine labs.

In conclusion, our case illustrates challenges in the management of obstetric patients experiencing acute hemorrhage in the setting of HELLP and DIC-induced coagulopathy. Involvement of multiple specialists is prudent to optimize the care of these challenging patients, although it is often not feasible in cases necessitating urgent fetal delivery. Thus, close communication between the anesthesiologist, surgeon, and intensivist is crucial in guiding intraoperative and ICU resuscitation efforts.

## Figures and Tables

**Table 1:** DIC score modified for pregnant women (adapted from Erez et al. [6]).

	Assigned score
*PT difference*	
<0.5	0
0.5–1	5
1.0–1.5	12
>1.5	25

*Platelets (×10* ^*9*^ * /L)*	
<50	1
50–100	2
100–185	1
>185	0

*Fibrinogen (g/L)*	
<3.0	25
3.0–4.0	6
4.0–4.5	1
>4.5	0

Combined score ≥ 26; sensitivity 88%; specificity 96%.

**Table 2:** Normal reference TEG values established for a parturient versus nongravid patient [26–30] (adapted from Katz et al. [30]).

	R-time^a^ (minutes)	K-time^b^ (minutes)	Alpha-angle (degrees)	Maximal amplitude (mm)	LY30^c^ (%)
Nongravid patient	6.7 (3.8–9.8)^*∗*^	2.0 (0.7–3.4)	62.3 (47.8–77.7)	60.6 (49.7–72.7)	1.2 (−2.3–5.7)
Term pregnancy	7.0 (1.0–13.0)	2.0 (0.2–3.8)	64.8 (47.6–82.0)	75.4 (64.6–86.2)	1.6 (0–8.8)

^a^R-time = reaction time, ^b^K-time = kinetics time, and ^c^LY30 = lysis 30 minutes after maximal amplitude. ^*∗*^Values are listed as mean with (95% CI).

## Data Availability

The laboratory data used to support the findings of this study are included within the article.
